# Osimertinib tolerance in a patient with Stevens Johnson syndrome during osimertinib therapy after treatment with pembrolizumab

**DOI:** 10.1186/s13223-023-00849-5

**Published:** 2023-10-28

**Authors:** Michael Lopez, Garo Hagopian, Linda Doan, Benjamin J. Lee, Nathan W. Rojek, Janellen Smith, Sai-Hong Ignatius Ou, Yesim Yilmaz Demirdag, Misako Nagasaka

**Affiliations:** 1 Division of Basic and Clinical Immunology, Department of Medicine, University of California, Irvine, USA; 2 Division of Hematology and Oncology, Department of Medicine, University of California, Irvine, USA; 3 Department of Dermatology, University of California, Irvine, USA; 4 Department of Pharmacology, University of California, Irvine, USA

## Abstract

**Background:**

Osimertinib has emerged as an important tool in the treatment of non-small cell lung cancers (NSCLC) with certain activating mutations of epidermal growth factor receptor (EGFR). However, Osimertinib may cause adverse effects, including severe cutaneous adverse reactions (SCARs) such as Stevens-Johnson syndrome (SJS) and toxic epidermal necrolysis (TEN). The risk of certain adverse effects may be increased in the setting of recent use of immune checkpoint inhibitor (ICI) therapy, although it is unclear whether recent use of ICI therapy is a risk factor for Osimertinib-induced SJS specifically.

**Case presentation:**

We present a patient with EGFR L858R mutation-positive metastatic NSCLC who developed Osimertinib-induced SJS after recent administration of eight cycles of a pembrolizumab-containing chemotherapy regimen. Osimertinib, which was the best treatment targeting his lung cancer, was avoided due to history of SJS. Four years later, because of unresponsiveness or side effects of alternative treatments, he underwent Osimertinib challenge and tolerated it.

**Conclusion:**

This case highlights the importance of multi-disciplinary care and supports the hypothesis that the risk of SJS to Osimertinib is significantly higher in the context of recent administration of ICI therapy and, patients may tolerate Osimertinib after certain time has elapsed after the last dose of ICI.

## Case report

Osimertinib, a third-generation epidermal growth factor receptor- tyrosine kinase inhibitor (EGFR-TKI), has emerged as an important treatment for non-small cell lung cancer (NSCLC). Compared to other EGFR-TKIs, Osimertinib has been associated with less severe and less frequent dermatologic side effects such as pruritus, xerosis, acneiform rash, paronychia, although rare cases of severe cutaneous adverse reactions (SCAR) have been reported [1–3]. The risk of adverse effects may be increased in the setting of recent use of immune checkpoint inhibitors (ICIs) such as pembrolizumab [4]. Here, we report a 63-year-old Vietnamese man who tolerated Osimertinib despite having experienced Stevens Johnson Syndrome (SJS) four years prior during Osimertinib treatment which was initiated 2 weeks after the last dose of an ICI, pembrolizumab.

**Fig. 1:**
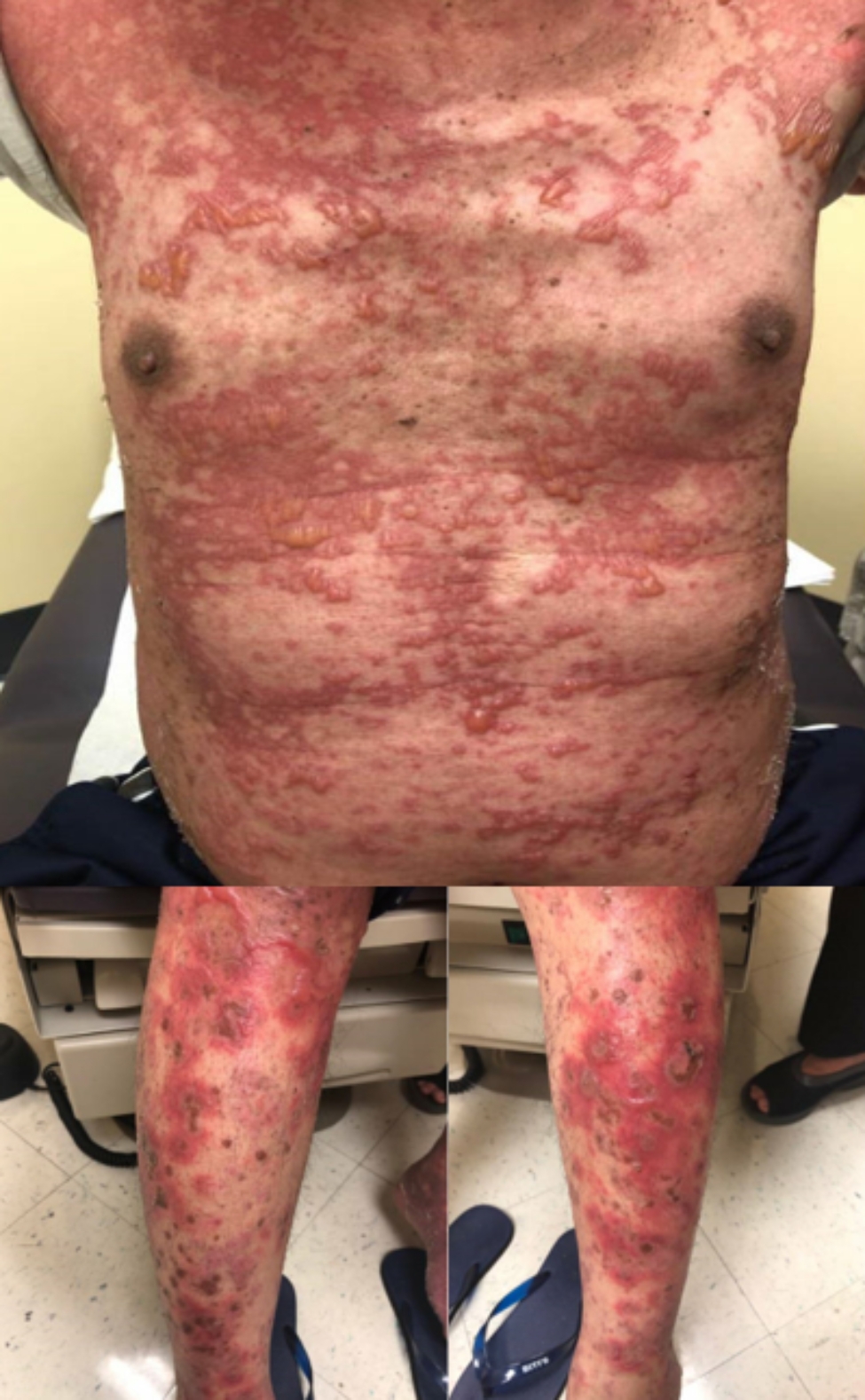
Osimertinib-induced SJS/TEN in a 63-year-old man with non-small cell lung cancer

**Fig. 2:**
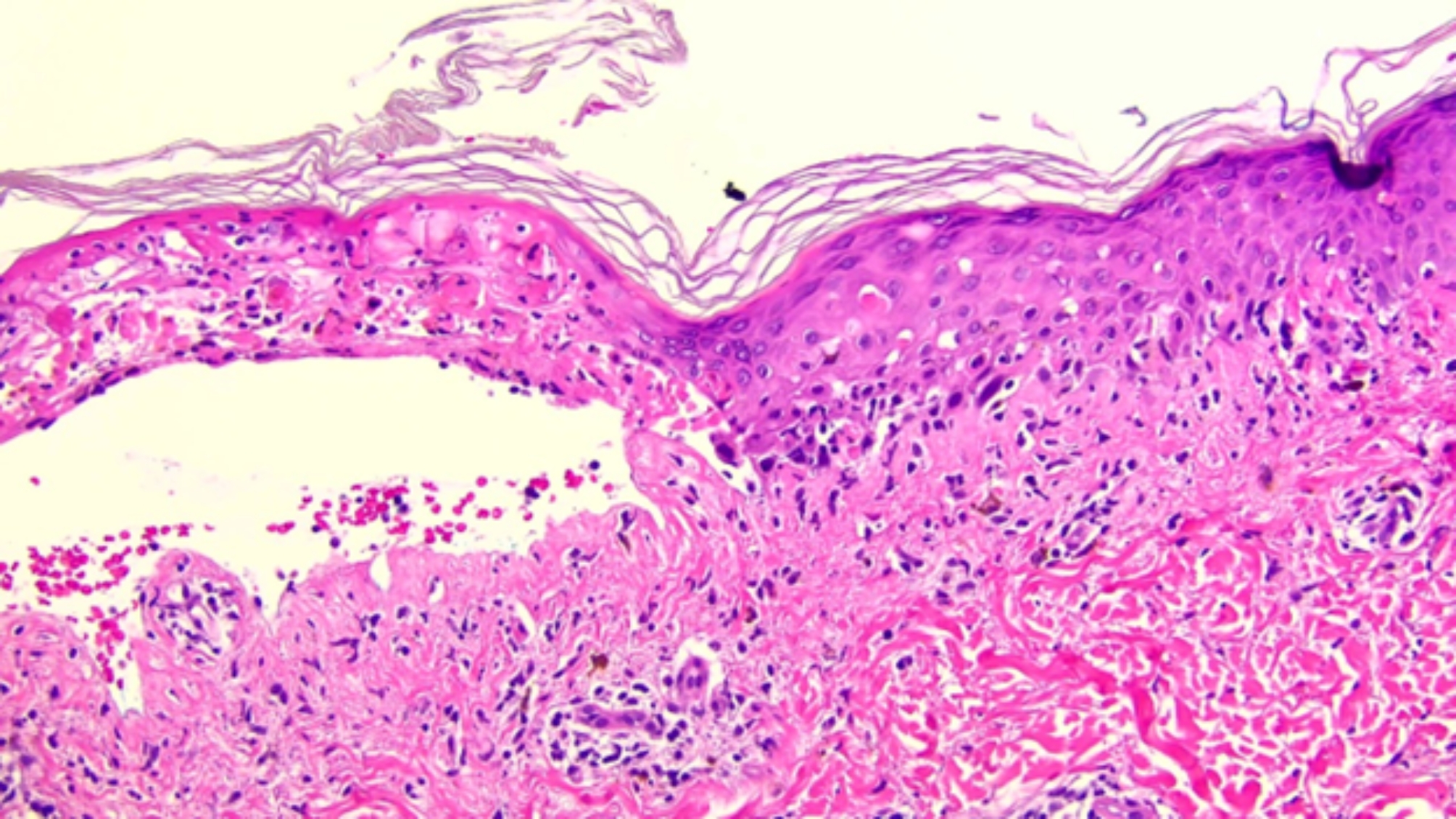
200x magnification. Skin biopsy demonstrating interface dermatitis with eosinophils, necrotic keratinocytes, and full-thickness epidermal necrosis, compatible with SJS/TEN in a 63-year-old man with non-small cell lung cancer

The patient was diagnosed with EGFR L858R mutation-positive NSCLC with bone metastasis and received 8 cycles of pemetrexed and pembrolizumab therapy. He was then transitioned to Osimertinib starting 2 weeks after the last cycle of pembrolizumab. However, 5 weeks after the initiation of Osimertinib he was hospitalized with diffuse skin eruption consistent with urticarial violaceous papules coalescing into plaques with tense bullae and erosions on his palms and soles (Fig. 1). He also developed sores on his lips and experienced painful swallowing and blurry vision. A skin biopsy demonstrated bullous eruption that was concerning for early SJS/TEN (toxic epidermal necrolysis) (Fig. 2). Osimertinib was discontinued and symptoms slowly resolved with supportive care. He received radiation, and then gemcitabine to control his cancer. Four years later, given the lack of suitable therapeutic options and disease progression, Osimertinib desensitization was attempted starting at 0.02 mg/day reaching the final dose of 80 mg in 25 days. However, 4 days after reaching 80 mg/day dose, patient developed erythematous papules and irritation feeling inside of his lips without any objective mucosal findings. Given concern for a possible early SJS, Osimertinib was discontinued, intramuscular methylprednisolone was administered, and the patient was prescribed a short course of prednisone. The rash and mucosal symptoms resolved within 2 weeks. His cancer, however, continued to progress, requiring radiation therapy for painful lymphadenopathy in the bilateral cervical and left axillary regions. Due to the mild reaction observed during the desensitization, and lack of treatment alternatives, five weeks after the last dose of Osimertinib, a shared decision was made for the patient to undergo an outpatient Osimertinib challenge. The starting dose was 40 mg daily reaching the goal dose of 80 mg daily over 5 weeks. At the time of the preparation of this manuscript, the patient has been tolerating Osimertinib 80 mg daily for approximately 6 months without any signs or symptoms of SJS or other SCARs.

## Discussion and conclusions

Significant immune-related adverse events such as pneumonitis have been reported during EGFR- TKI treatment. Osimertinib, a third generation EGFR-TKI, is associated with less severe and less frequent adverse events although the risk is increased after exposure to ICIs [4]. Osimertinib tolerance was reported about 8 months after the development of pneumonitis during Osimertinib treatment which was preceded by an ICI therapy in a patient with lung cancer [5]. Interestingly, successful Osimertinib rechallenge have also been observed in 8 patients who developed Osimertinib-induced pneumonitis and in 1 patient who developed Osimertinib-induced hepatotoxicity without prior ICI therapy [6, 7]. According to these studies, in patients who experienced Osimertinib-induced pneumonitis, the median interval from the first Osimertinib course and Osimertinib re-challenge was 6.3 months. In the case of Osimertinib-induced hepatotoxicity, Osimertinib was restarted at 8 mg/day which was considered as desensitization dose, about 5 weeks after it was discontinued due to worsening of liver function. These reports suggest that the duration between treatments may play a role in tolerability of Osimertinib re-challenge.

SCARs associated with Osimertinib with or without prior ICI exposure have been reported in only a few cases [2, 3, 8, 9]. Successful reintroduction of Osimertinib in our patient may suggest that prior ICI therapy contributed to the development of SJS during Osimertinib treatment. However, we cannot exclude the potential contribution of other factors, such as the time elapsed (four years) since the initial reaction. To our knowledge, this is the first successful reintroduction of Osimertinib in a patient with a remote history of Osimertinib-induced SJS in the setting of recent ICI therapy.

## Data Availability

All materials are included in this article.
